# Brain-consistent architecture for imagination

**DOI:** 10.3389/fnsys.2024.1302429

**Published:** 2024-08-20

**Authors:** Hiroshi Yamakawa, Ayako Fukawa, Ikuko Eguchi Yairi, Yutaka Matsuo

**Affiliations:** ^1^School of Engineering, The University of Tokyo, Tokyo, Japan; ^2^The Whole Brain Architecture Initiative, Tokyo, Japan; ^3^Graduate School of Science and Technology, Sophia University, Tokyo, Japan

**Keywords:** imagination, function-oriented structure-constrained interface decomposition method, reverse engineering, artificial intelligence, brain-inspired software

## Abstract

**Background:**

Imagination represents a pivotal capability of human intelligence. To develop human-like artificial intelligence, uncovering the computational architecture pertinent to imaginative capabilities through reverse engineering the brain's computational functions is essential. The existing Structure-Constrained Interface Decomposition (SCID) method, leverages the anatomical structure of the brain to extract computational architecture. However, its efficacy is limited to narrow brain regions, making it unsuitable for realizing the function of imagination, which involves diverse brain areas such as the neocortex, basal ganglia, thalamus, and hippocampus.

**Objective:**

In this study, we proposed the Function-Oriented SCID method, an advancement over the existing SCID method, comprising four steps designed for reverse engineering broader brain areas. This method was applied to the brain's imaginative capabilities to design a hypothetical computational architecture. The implementation began with defining the human imaginative ability that we aspire to simulate. Subsequently, six critical requirements necessary for actualizing the defined imagination were identified. Constraints were established considering the unique representational capacity and the singularity of the neocortex's modes, a distributed memory structure responsible for executing imaginative functions. In line with these constraints, we developed five distinct functions to fulfill the requirements. We allocated specific components for each function, followed by an architectural proposal aligning each component with a corresponding brain organ.

**Results:**

In the proposed architecture, the distributed memory component, associated with the neocortex, realizes the representation and execution function; the imaginary zone maker component, associated with the claustrum, accomplishes the dynamic-zone partitioning function; the routing conductor component, linked with the complex of thalamus and basal ganglia, performs the manipulation function; the mode memory component, related to the specific agranular neocortical area executes the mode maintenance function; and the recorder component, affiliated with the hippocampal formation, handles the history management function. Thus, we have provided a fundamental cognitive architecture of the brain that comprehensively covers the brain's imaginative capacities.

## 1 Introduction

Imagination is the ability to generate patterns that differ from reality, utilizing representational elements that correspond to elements in the environment acquired through experience. For intellectual systems, imagination is crucial, as it is necessary for generating hypotheses for situations that cannot be directly experienced. Furthermore, imagination forms the foundation of all meaning, understanding, and inference (Johnson, 2013). It is believed that the imaginative capabilities present in modern humans were acquired during the “Great Leap Forward,” approximately 70,000 years ago (Diamond, 1992). Given that imagination is a core function in intelligence, designing an architecture for imagination is highly beneficial for developing brain-like artificial intelligence and for the computational understanding of the brain. Here, the term “architecture” refers to a description of a system in which multiple computationally meaningful components are organically connected, thereby forming the design information for the software.

Since the beginning of this century, cognitive pathology research has progressed, particularly concerning imagination. For example, the lack of creativity in children with autism and Asperger's syndrome has been studied (Craig and Baron-Cohen, 1999). Additionally, a comprehensive analysis of pathological findings suggests that social imagination, particularly as embodied in the default mode network of the human brain, may mediate representations along the opposite dimensions of autism and risk for psychosis/emotional disorders (Crespi et al., 2016). Several types of studies measuring brain neural activity have been conducted. EEG analysis has shown that brain waves appear to be specific to visual imagination and perception, especially in the alpha frequency band (Xie et al., 2020). An fMRI study capturing the default mode network of human imagination of the future demonstrated that the medial temporal lobe responds to the vividness of the imagined event, whereas the dorsal or core Default Mode Network responds to its semantic depth (Lee et al., 2021). When a motor schema is established by motor imagery learning, interregional connectivity in the occipital lobe may be significantly reduced, as demonstrated by fMRI studies (Zhang et al., 2012). While such studies do not directly propose an architecture, they can provide insight into its design. From a human-focused cognitive neuroscience approach, some studies conceptualize the function of imagination by estimating the role of brain organs associated with mental disorders. Imagination has been conceptualized as an important mediator between acquired knowledge and creative insight, constraining potential solutions through mental simulation or “incubation” (Duch, 2007). Moreover, visual imagery has been hypothesized to involve a network of brain regions from the frontal to the sensory cortex, and imagination has been proposed to function similarly to a weaker version of afferent perception (Pearson, 2019). However, these studies offer primarily phenomenological explanations and are somewhat distant from architectural design; moreover, they did not consider brain organs associated with imagination other than the neocortex and hippocampus.

Agnati et al. (2013) proposed a hypothesis on the mechanisms involved in the executive function of imagination; however, this study focused only on neocortical mechanisms. Based on their considerations, Marques and Holland (2009) defined an architecture that realizes the conditions for functions performed by the imagination and implemented it in a humanoid simulated robot and a real robot. This study focuses primarily on the neocortex and briefly describes the hippocampus. As described above, studies of architectures of imagination tend to focus solely on the neocortex, with little consideration of other connected brain organs and their functional roles.

Conversely, a research area related to imagination is the study of consciousness in computational neuroscience, as described below. Many studies relate phenomenal features of neural activity to the anatomical structure of brain regions, as follows: neural field theories (Kinsbourne, 1988) assume that consciousness is a characteristic of a specific pattern of neuron firing. The field model (Pockett, 2000; McFadden, 2002) assumes that consciousness is related to the electromagnetic field of the brain. The thalamocortical rhythms model (Llinás et al., 1998) assumes that neural activity phenomena in the thalamocortical loop create the state of consciousness. Interconnected neural networks (Crick and Koch, 2003) assume that “coalitions” of neurons determine the content of consciousness. In theories such as the information integration theory (Tononi, 2004), information closure theory (Chang et al., 2019), and higher-order thinking theory (Rosenthal, 1986), the aforementioned core function is considered to be realized by the coordination of the neocortex and thalamus. Global workspace theory describes consciousness as a process that leads information, selected by attention from the realm of unconsciousness, into the global workspace and flexibly processes and retains it (Baars, 1993). Moreover, the global workspace dynamics theory (Baars et al., 2013) incorporates the idea of the dynamic core hypothesis (Edelman and Tononi, 2000), which corresponds to the functions of the neocortex and thalamus. Additionally, Francis Crick posited that the claustrum is the neural foundation of consciousness and plays the role of a conductor in the orchestra of the neocortex (Crick and Koch, 2005; Stevens, 2005).

As noted earlier, the research on imagination and consciousness has been centered on the phenomena and computational functions associated with the neocortex. The neocortex, widely regarded as a repository for a diverse array of representations, is theorized to play a pivotal role in the execution of imagination through the integration of these representations. Nonetheless, drawing from previous studies, other brain structures, including the hippocampus, thalamus, and prefrontal cortex, also contribute to these processes. Therefore, designing a comprehensive architecture for imagination incorporating these peripheral brain components is necessary.

A notable approach for designing architecture that emphasizes consistency with the brain's anatomical structure based on neuroscience findings is the Structure-Constrained Interface Decomposition (SCID) method (Yamakawa, 2021). The SCID method is a reverse engineering process, focusing primarily on the brain's anatomical structure by identifying the Regions of Interest (ROI) in the brain related to the function of interest. However, a current challenge of the SCID method is its inapplicability to broad brain regions, due to the requirement of a detailed understanding of the anatomical structure of the ROI down to the mesoscopic level. Imagination is a complex ability that involves a wide range of brain regions; the current SCID method cannot be used to design architecture that adequately supports it.

Therefore, in this study, we aimed to propose a new function-oriented SCID method, comprising four distinct steps, that enables the reverse engineering of a broad spectrum of brain regions. We have also applied this innovative method to elucidate the computational architecture underpinning the imaginative capability of the brain, resulting in a proposed architecture involving five key brain structures.

## 2 Methods

This section first describes the Brain Reference Architecture (BRA)-driven development approach. Brain Reference Architecture is abbreviated here as BRA. Subsequently, the SCID methodology, which implements the design portion of BRA-driven development, is detailed. Based on this, we propose a function-oriented SCID method that can improve the weaknesses of the conventional SCID method.

### 2.1 BRA-driven development

Brain Reference Architecture (BRA)-driven development is a methodology conceived to construct brain-like software. This methodology emerged as a response to three challenges previously encountered in the development of brain-inspired software: (Challenge 1) Professionals adept in both neuroscience and software development are extremely rare, and cultivating such interdisciplinary expertise is challenging and complex. (Challenge 2) The vast body of neuroscience knowledge covering the entire brain makes it impractical to rely on an individual's cognitive capacity for designing software that seamlessly integrates all brain functions. (Challenge 3) An appropriate level of granularity has to be selected in the referenced brain descriptions to faithfully represent the brain's cognitive functions in software.

**Figure 1:**
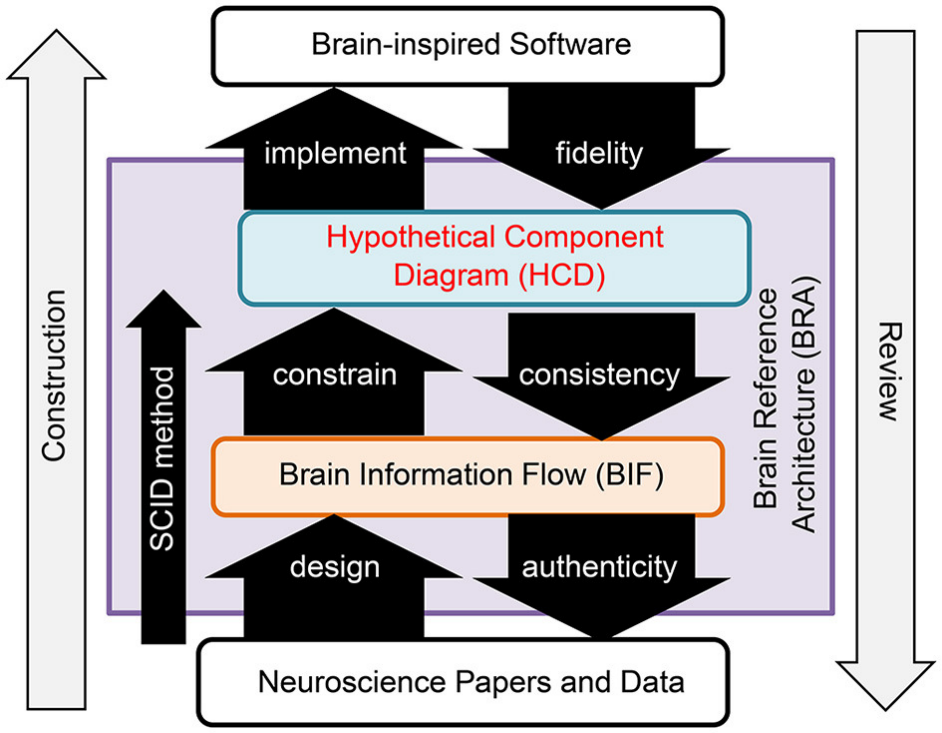
Brain reference architecture-driven development.

BRA-driven development addresses Challenge 1 by dividing the process into two distinct phases: design of the BRA, which serves as the specification information for brain-inspired software, and implementation of the software according to this BRA. Moreover, this process overcomes Challenge 2 by promoting collaborative work, facilitated through the standardization of the BRA data (Figure 1).

BRA is a standardized data format that functions as the specification information for brain-like software. It encompasses data representing the brain's information flow and a hypothetical component diagram articulated in conjunction with this data.

- Brain Information Flow (BIF) data: This data refers to the information flow of the brain's neural circuits at a mesoscopic anatomical level. It is formatted as a directed graph, with diverse granularity “circuits” within the brain represented as nodes and “connections,” corresponding to axon projections between them, depicted as links. Notably, the origins of these connections are constrained to Uniform Circuits.
- Hypothetical Component Diagram (HCD) data: This constitutes a hypothetical component diagram that aligns functions with the anatomical structure of the Top Level Function (TLF) executed by the Region of Interest (ROI) within the BIF. Importantly, an HCD can correspond to any circuit within the BIF, and multiple HCDs can overlap and be associated with a specific circuit in the BIF.

Challenge 3 was resolved by defining the smallest circuit unit to be portrayed in the Binary Interchange Format (BIF) as the Uniform Circuit. The Uniform Circuit is, fundamentally, an ensemble of neurons composed of a specific cell type situated within a designated brain region.

The creation of the BRA, depicted on the left side of Figure 1, adheres to a precise procedure known as the SCID method. This reverse engineering method entails an in-depth analysis of the anatomical structures within an ROI and hypothesizes a functional system that could realize the top-level function that the ROI serves in alignment with its structure. The BRA was evaluated in a phased manner, as illustrated on the right side of Figure 1. The evaluation includes a fidelity assessment, which evaluates the alignment of the software with the HCD; a consistency evaluation, which verifies the inclusion of the structure utilized in the HCD within the BIF structure; and an authenticity evaluation, which verifies whether neuroscience findings substantiate the BIF.

### 2.2 Conventional SCID method

The SCID method is performed by creating BRA data.

The SCID method involves three steps:

- Step 1. BIF construction: Investigating brain anatomy and neural circuits in the ROI to define BIF.
- Step 2. Alignment of ROI and TLF: Identifying the Top-Level Function (TLF).
- Step 3. HCD design: Candidate component diagrams describing computational functions and interfaces are enumerated from the HCDs, and inappropriate ones are eliminated based on neuroscientific evidence and logical consistency. The remaining ones are HCDs.

As neuroscience has now accumulated anatomical knowledge of a relatively wide range of regions, the SCID method uses this knowledge to design functions, resulting in broad applicability.

This SCID method eases the process of HCD design by detailing the BIF within the ROI of interest to a uniform circuit granularity. However, this approach presents challenges when utilized to reverse engineer extensive regions of the brain's computational functionality. Particularly, generating a BIF for a broad region in the initial step is a complex task. Moreover, even if a BIF is successfully build, deconstructing a high-level abstract TLF into uniform circuit granularity functionalities for a wide range of ROI in the third step is also challenging.

### 2.3 Proposing the function-oriented SCID method

Based on the aforementioned technical background, a new methodology called the “function-oriented SCID method” was proposed to determine the TLFs of multiple small ROIs from highly abstract functions in a broad ROI.

The proposed methodology consists of the following four steps.

- Step 1. Capability and requirements definition: Define a Broad TLF at a high level of abstraction in an expansive brain region (e.g., whole brain) and decompose it into a set of requirements to realize it.
- Step 2. Function decomposition: Build a set of independent functions that can satisfy all requirements and significant biological constraints.
- Step 3. Architecture design: Design a mechanism to realize each function as a component that defines input/output signals with semantics. Ensure that the created components work cohesively to meet all requirements.
- Step 4. Brain region mapping: Map the above components to reasonable brain regions concerning input/output signal semantics and internal processing feasibility. However, if no appropriate mapping is found, return to Step 2 and revisit the decomposition of the function.

To facilitate Step 2, TLF specifications and biological constraints are considered to narrow down the candidate functional decompositions.

In step 4, the input and output signals required for each component are considered during the solution to see if the inputs and outputs can be mapped to candidate brain regions, considering the semantics of the signals. The feasibility of internal processing of the component in the candidate brain regions is also assessed if possible. If, after these considerations, a consistent mapping is possible, the brain region is mapped to the component.

**Figure 2:**
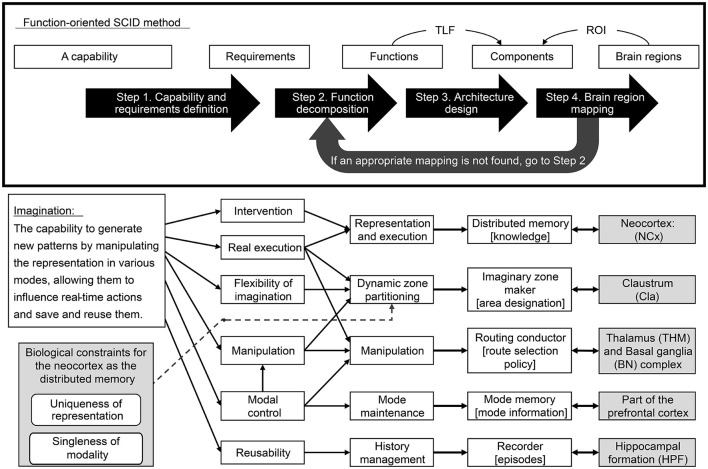
Function-oriented SCID method and its application for imagination capability.

As mentioned above, the process advances through Steps 2, 3, and 4. If mapping to brain structures fails during Step 4, the process reverts to Step 2 (see Figure 2). To optimize efficiency, it's crucial to minimize such revisions. Consequently, it's advisable to perform functional decomposition in Step 2 while anticipating the subsequent mapping to brain structures as much as possible.

Therefore, for each specific component, the TLF is defined in Step 2, and the ROI is identified in Step 4. By applying the conventional SCID method to these ROI/TLF pairs, a hypothesis of the computational function of the brain can be constructed at a more detailed level within the ROI.

## 3 Applying the function-oriented SCID method for imagination

In this section, we apply the functionally oriented SCID method to the brain's imagination according to the four steps of this method.

A comprehensive illustration of the approach is shown in Figure 1. In Section 3.1, as the first step, we set the TLF in imagination while considering the treatment of modalities and decompose it into six requirements. In Section 3.2, as the second step, we organize the TLFs into six functionalities based on the requirement specifications, considering the biological constraints for the neocortex as distributed memory. In Section 3.3, as the third step, we construct an architecture for imagination by designing five components to realize the functions. In Section 3.4, as the fourth step, we propose an architecture for creativity in the brain by mapping each component to a brain region.

### 3.1 Step 1. Capability and requirements definition: define imagination capability

#### 3.1.1 Modes that govern imagination

Representational patterns that humans can obtain directly from the environment involve the contexts wherein their own experience occurs. In contrast, various imaginary contexts deviate from them in some respect. For instance, recalling a previous memory is associated with the context of “the past.” In the case of a newly generated pattern that is made from a combination of representations, its context is not self-explanatory. However, if this pattern represents a prediction, it assumes the context of “the future.” If it guides subsequent actions, it is regarded as an “intention.” Here, “intention” refers to the mental process of planning a specific course of action (Bratman and Others, 1987).

Modal logic (Garson, 2018) is a well-known theoretical background for understanding how contexts can modify the patterns they encompass. Based on modal logic, we defined a context that modifies the entirety of an imagined pattern as a “mode.” Individuals frequently engage in social reasoning by adopting the perspectives of others, although modal logic in the realm of artificial intelligence does not usually account for this. Therefore, in this study, the mental states of others other than the self shall also be treated as “modes” of some form.

The treatment of modals in imagination has received much attention in philosophy. Still, much of the work seems to focus on epistemological arguments regarding the range of beliefs that are legitimate to imagine (Kung, 2010; Ichikawa and Jarvis, 2012).

For higher animals, including humans, the ability to infer others' beliefs and intentions is a critical requirement for survival in a society consisting of members of the same species. As noted in the study of the mind theory, humans acquire the ability to estimate the beliefs and intentions of others in early childhood. It is also known that the brain contains mirror neurons (di Pellegrino et al., 1992) which react to the actions of others as if they were one's own actions.

In order to exert imaginative abilities, modes are indispensable for distinguishing the present, the past and the future, the self and the others, beliefs, and intentions. This idea has been debated in the field of philosophy (Nichols, 2006; Kind, 2016), but has been largely overlooked in both neural processing technologies and computational neuroscience to date.

From our perspective, the following provides a more specific explanation of the necessity for apprehending and managing modes regarding imagined patterns.

First, the process of imagining differs depending on the type of mode. For instance, future prediction would manipulate representations from the present to the future, inference about the past would manipulate representations by reversing causation from the present to the past, and performing obligatory actions would manipulate the future image with the goal of realizing a state wherein the obligation has been fulfilled. Second, the manner of utilizing patterns depends on their mode. For instance, if an agent conceives a pattern in the mode of intention about its own future, that pattern should be acted on when an appropriate circumstance is found. Conversely, if the agent conceives a pattern that involves the mode of prohibition, which is a kind of obligation that dictates not to do something, that pattern should deter action, regardless of circumstances. Third, when verbalizing imagined patterns, their modes affect the conjugation of auxiliary verbs and main verbs. Fourth, storing a pattern must be paired with the current mode state. A pattern being restored at a later time with an incorrect mode would cause a situation corresponding to the symptom of memory confusion.

#### 3.1.2 Definition of imagination

Imagination has been studied across various fields, including neuroscience, philosophy, psychology, and artificial intelligence. Each discipline has approached the concept of imagination from its unique perspective, leading to a diverse array of definitions. These definitions often reflect the specific focus and methodologies employed within each field. Neuroscientists, for instance, may emphasize the neural mechanisms underlying the formation of mental images, while philosophers might explore the representational nature of imagination and its relation to other cognitive processes. Psychologists, on the other hand, could focus on the role of imagination in mental simulation and its impact on behavior and decision-making. Researchers in artificial intelligence may view imagination as a crucial component in creating intelligent systems capable of generating novel ideas and solutions. The following list presents a selection of definitions that showcase the breadth of perspectives on imagination:

- To imagine is to represent without aiming at things as they actually, presently, and subjectively are. One can use imagination to represent possibilities other than the actual, to represent times other than the present, and to represent perspectives other than one's own. Unlike perceiving and believing, imagining something does not require one to consider that something to be the case. Unlike desiring or anticipating, imagining something does not require one to wish or expect that something to be the case (Liao and Gendler, 2020).
- Imagination is the production or simulation of novel objects, sensations, and ideas in the mind without any immediate input of the senses (Wikipedia contributors, 2023).
- Imagination, considered as “forming new ideas, mental images, or concepts,” represents a central facet of human social evolution and cognition (Crespi, 2016).
- Imagination may depend on separate neural networks involved in the construction and evaluation of imagined future events (Lee et al., 2021).
- What is the human imagination? What is this amazing ability, which most of us have, that allows us to travel through space and time, testing out different virtual worlds, objects, foods, fears, and pleasures (Pearson, 2019)?
- Imagination: the act or power of forming mental images of what is not actually present or has never been actually directly experienced. Notably, imagination not only has the potential to enrich the meaning of an experience and deepen understanding by multiplying and expanding the perspectives from which a phenomenon can be considered, but it also allows anticipating the outcome of an action without actually performing it via a “simulation” process. At its peak, imagination is the very mental faculty underlying visionary and creative thought (Agnati et al., 2013).
- Imagination can be defined broadly as the manipulation of information that is not directly available to an agent's sensors (Marques and Holland, 2009).
- Imagination involves episodic memory retrieval, visualization, mental simulation, spatial navigation, and future thinking, making it a complex cognitive construct (Jung et al., 2016).

Based on the above definitions, the common denominator of imagination is the “capability to generate new patterns by manipulating the representation,” which is also used in this paper. However, the manipulation must be mode-dependent, as previously described (Section 3.1.1); therefore, “depending on various modes” is added. Furthermore, as this study deals with the architecture of the imagination, it is necessary to consider the peripheral capabilities along with the core capability of the imagination. The peripheral capability of imagination includes the capability to store and reuse the results of imagination and reflect them in real-time actions. For this reason, we added “allowing them to influence real-time actions and save and reuse them” to the definition.

Based the previous considerations, this paper defines imagination as follows.

##### Definition of Human Imagination:

The capability to generate new patterns by manipulating the representation depending on various modes, allowing them to influence real-time actions and save and reuse them.

#### 3.1.3 Requirements of imagination

First, we took the possible requirement specifications from the above definition and organized them into six categories.

- **Intervention requirement:**
  The requirement to make the outcome of an execution (patterns) affect real-time execution (behavioral output to the environment and internal attention control). For example, a plan that is built by imagination has to be put into action at the right time.
- **Real-execution requirement:**
  Apart from imaginative abilities, continuously performing real-time processing grounded in the environment using representations in the distributed memory is essential for maintaining activities as an agent. This requirement is primarily related to the ideomotor theory in psychology (James, 1890; Shin et al., 2010), which posits that unconscious mental images or thoughts can automatically trigger corresponding motor actions or behaviors.
- **Flexible imagination requirement:**
  The requirement to combine representations widely dispersed in the distributed memory to create various patterns flexibly.
- **Manipulation requirement:**
  The requirement to imagine new patterns by manipulating representations in the distributed memory, depending on the types of their mode. Note that the imagined patterns do not directly affect the actions of the intelligent system.
- **Modal control requirement:**
  The requirement to generate a series of imagined patterns under a particular mode.
- **Reusability requirement:**
  The requirement to preserve the outcome of imaginative generation (patterns) and its modes together in order to retrieve and reuse them at a later time.

The previously defined human imaginative capacity is realized through multiple above requirements that operate in concert. Firstly, the aspect of “manipulating the representation depending on various modes” is augmented by two requirements: Mode Control and Manipulation. Mode Control offers the ability to produce a sequence of imaginative patterns under specific conditions or aspects, whereas Manipulation bestows the capability to create new patterns by manipulating representations. Secondly, the notion of “generating new patterns” is underpinned by the requirement for Flexibility of Imagination. This necessitates flexibly combining a wide range of representations stored in distributed memory, thereby generating diverse patterns. Thirdly, the facet of “influencing real-time actions” is embodied by two requirements: Intervention and Real Execution. An intervention allows the generated imaginative patterns to be reflected in real-time external behavior or internal attention control. Real Execution fortifies this aspect by enabling continuous real-time processing as an agent, thereby facilitating interaction with the external world. Lastly, the aspect of “save and reuse them” is complemented by the requirement for Reusability. This involves storing the generated imaginative patterns and their mode information for future reuse.

### 3.2 Step 2. Functional decomposition: functions for imagination capability

The second step of the function-oriented SCID method involves organizing the above requirement specifications into five functions. In doing so, we introduced a dynamic zone segmentation function, considering the significant biological constraints of the neocortex, which functions as a distributed memory.

#### 3.2.1 The necessity of dynamic zone partitioning function in the neocortex

The neocortex corresponds to distributed memory in the brain and is classified into numerous areas such as Broadman's brain map. Although the neocortex has an almost common mechanism, each area handles different information related to the senses, such as vision and hearing, motor control, language, and thought. In the brain, the ability to flexibly combine information stored in different neocortical areas, as described below in Section 3.4.3, is the key to rich human intellectual abilities. In addition, its function is the executive function of both Real and Virtual, as described above.

To proceed with the discussion, we first point out that the neocortex, as a distributed memory mechanism, has two biological properties that differ from those of distributed memory on the computer.

- Uniqueness of representation :
  It may be postulated that individual distributed representations within the neocortex exhibit unique responsiveness characteristics. This phenomenon is attributable to the intrinsic capacity of neurons to modify their response properties through learning processes. The absence of a neurological mechanism for replicating identical response properties across disparate representations further substantiates this assertion. Although neurons situated in proximal local clusters may manifest similar responsiveness when exposed to analogous stimuli, it is advisable to regard representations that are geographically distant from one another as inherently distinct.
- Singleness of modality:
  It appears that, to a single neuron or local neural circuit, only one state of mode can be associated at a given point in time. This supposition is based on the intuition that, if there were a group of 100 neurons representing a “cat,” they would be challenging to code, as 80 of them represent a cat that is currently and visibly present, while the remaining 20 neurons represent a cat that was seen yesterday.

The representations in the distributed memory are obtained by Representation and Execution (RAE) and utilized for execution. Since each of these representations in the distributed memory exhibits uniqueness as a characteristic, exerting imaginative abilities requires a mechanism that enables the flexible use of various representations while satisfying the flexible imagination requirement to divert representations in distributed memory. However, since a representation can be associated with only one mode, the only way of diverting its use is to use it at a different time. For this reason, a function that dynamically sets an area for imagination (hereinafter called the imaginary zone) has to be introduced in a distributed memory with properties similar to the neocortex.

The RAE and Manipulation functions are required in the distributed memory's real and imaginary zones. These two areas would correspond to System 1 and System 2 in the double-process theory (Stanovich, 2009) and to the immediate response and contemplation in cognitive architecture.

Patterns on a distributed memory should be treated differently according to their modes (see Section 3.1.1). For this reason, the imaginary zone, to which various modes can be given, needs to be clearly separated from the real zone. The dynamic zone partitioning function accomplishes this separation, satisfying the requirement of mode maintenance, and enabling the imagination of a series of patterns in the imaginary zone under a particular mode.

The following scientific findings also enhance the potential of the proposed idea. There is some evidence that imagination is like a weak form of perception in the neurophysiology involved in vision (Ishai and Sagi, 1995; Pearson et al., 2008; Pearson, 2019). Imagination is also thought to be the capability to reuse the neural structures originally involved in the execution of a function for an image of the virtual execution of that function (Agnati et al., 2013).

Moreover, similar ideas to the dynamic zone partitioning function have been proposed, as seen in the dynamic core hypothesis (Edelman and Tononi, 2000), and in the global workspace dynamics inspired by the former theory (Baars et al., 2013). This function is sometimes referred to as a “cognitive decoupling” in a cognitive science field (Perner, 1991; Tooby and Cosmides, 2000; Nichols and Stich, 2003).

#### 3.2.2 Functions derived from requirements

The functions that realize the six requirements, enumerated above, are identified while taking into account a biological constraint of the neocortex as a distributed memory to reveal that the five functions shown in Figure 1 are necessary.

- **Representation and execution (RAE) function:**
  Functions to represent different types of information. There are sensory representations to execute perception, motor representations to execute control, and higher-order representations to execute thought. These functions can be divided into those that output actions in real-time to the environment and those that are imaginative and do not contribute directly to actions. However, functions that reflect the results of imagination in real-time behavior must also be included to meet intervention requirements.
- **Dynamic zone partitioning function:**
  This function dynamically specifies an imaginary zone in distributed memory and maintains it for a necessary period. An imaginary zone is a subset of the representation for executing imagination on distributed memory. This functionality allows for manipulation and flexible imagination requirements Note that the complementary set of the imaginary zone will be referred to as the real zone.
- **Manipulation function:**
  This function imagines new patterns by manipulating representations on the distributed memory according to a policy appropriate to its type of mode and context to meet the requirements of manipulation and modal control.
- **Mode maintenance function:**
  This function maintains the current mode that affects the sequence control of patterns generated or replayed on the distributed memory to meet the requirements of mode control.
- **History management function:**
  This function records the pattern of activity in distributed memory at a given time as an episode. It restores patterns of activity in distributed memory from episodes as needed. Patterns include mode, context, routing, and designation control.

#### 3.2.3 Requirements realized by functions

Next, it is necessary to verify that all requirements are met by fulfilling the functions derived above. In the following, we will review this point for each of the six requirements.

- **Intervention requirement:**
  This requirement is fulfilled by the “function to reflect the results of imagination in real-time behavior,” which is included in the RAE function.
- **Real-execution requirement:**
  This requirement is achieved by outputting the results calculated by the Manipulation function in the real zone on Distributed Memory, which has a Representation and execution function, partitioned by the Dynamic-zone partitioning function, as the results of real-time processing.
- **Flexible imagination requirement:**
  This requirement is fulfilled by flexibly setting the area on Distributed Memory used for imagination through the role ofthe Dynamic-zone partitioning function.
- **Manipulation requirement:**
  Distributed Memory, designated by the Dynamic-zone partitioning function, through the Manipulation function.
- **Modal control requirement:**
  This requirement is accomplished by generating a pattern by the Manipulation function based on the mode maintained by the Mode maintenance function.
- **Reusability requirement:**
  This requirement is accomplished by the History management function, which stores an index of activity patterns in distributed memory and returns the results to reproduce those patterns in distributed memory as needed.

### 3.3 Step 3. Architecture design: architecture for imagination

In the third step of our design process, we devised an architecture comprising five key components: routing conductor, mode memory, imaginary zone conductor, recorder, and distributed memory-which corresponds to the neocortex. These components were integrated into a hypothetical architecture, represented as a component diagram that defines the meaning of the signals exchanged between them (see Figure 2). This section substantiates that each function is realized through coordination among these components. The meaning of the signals exchanged between the components will be explained in the received components.

- **Distributed memory** (and its role in RAE function):
  The distributed memory component is a highly versatile, distributed associative storage system that excels at representing and manipulating various patterns. This component outputs real-time action signals in response to environmental sensing signals. The various internal pattern information is distributed to the surrounding components. This component dynamically sets imaginary zones based on area designation signals, changes associative behavior according to transmission channel signals, and reproduces patterns according to pattern index signals.
- **Imaginary zone maker** (and its role in dynamic zone partitioning function):
  The imaginary zone maker is a component that realizes the dynamic zone partitioning function. The designation policy generates area designation signals that specify areas of imaginary zones on distributed memory based on the received designation control signals.
- **Routing conductor component** (and its role in the manipulation function):
  The Routing Conductor component is the mechanism responsible for the manipulation function. The route selection policy chooses and generates the appropriate transmission channel signals from this component's received routing pattern signals. This selection is controlled according to the type of mode obtained from the current mode signal, depending on the context information by the received context signal.
- **Mode memory** (and its role in mode maintenance):
  The mode memory holds the specific mode pattern signal obtained from one of the distributed memory as mode information and transmits it continuously as a current mode signal. This mechanism enables the mode maintenance function.
- **Recorder component** (and its role in history management):
  The recorder component serves as the backbone of the history management function. This component records the episodes as indexes^1^ of acquired patterns. These episodes restore and send to distributed memory when needed. Here, pattern signals encompass mode, context, routing, and designation control.

The architecture proposed above shows that the five functions discussed in the previous subsection can be realized mainly by the components corresponding to each of them.

### 3.4 Step 4. Brain region mapping: imagination architecture in the brain

As the fourth step, we propose a brain-imagination architecture as a finishing touch to the “function-oriented SCID method” by mapping each component to a part or component of the brain. In this study, we envision assigning the neocortex to distributed memory. Therefore, we align appropriate brain regions to Imaginary zone makers, routing conductors, mode memory, and recorder. Assigning a brain region to each component would set the TLF at a relatively small ROI. Therefore, the results can be used for reverse engineering through the conventional SCID method based on the TLFs of each brain region.

**Figure 3:**
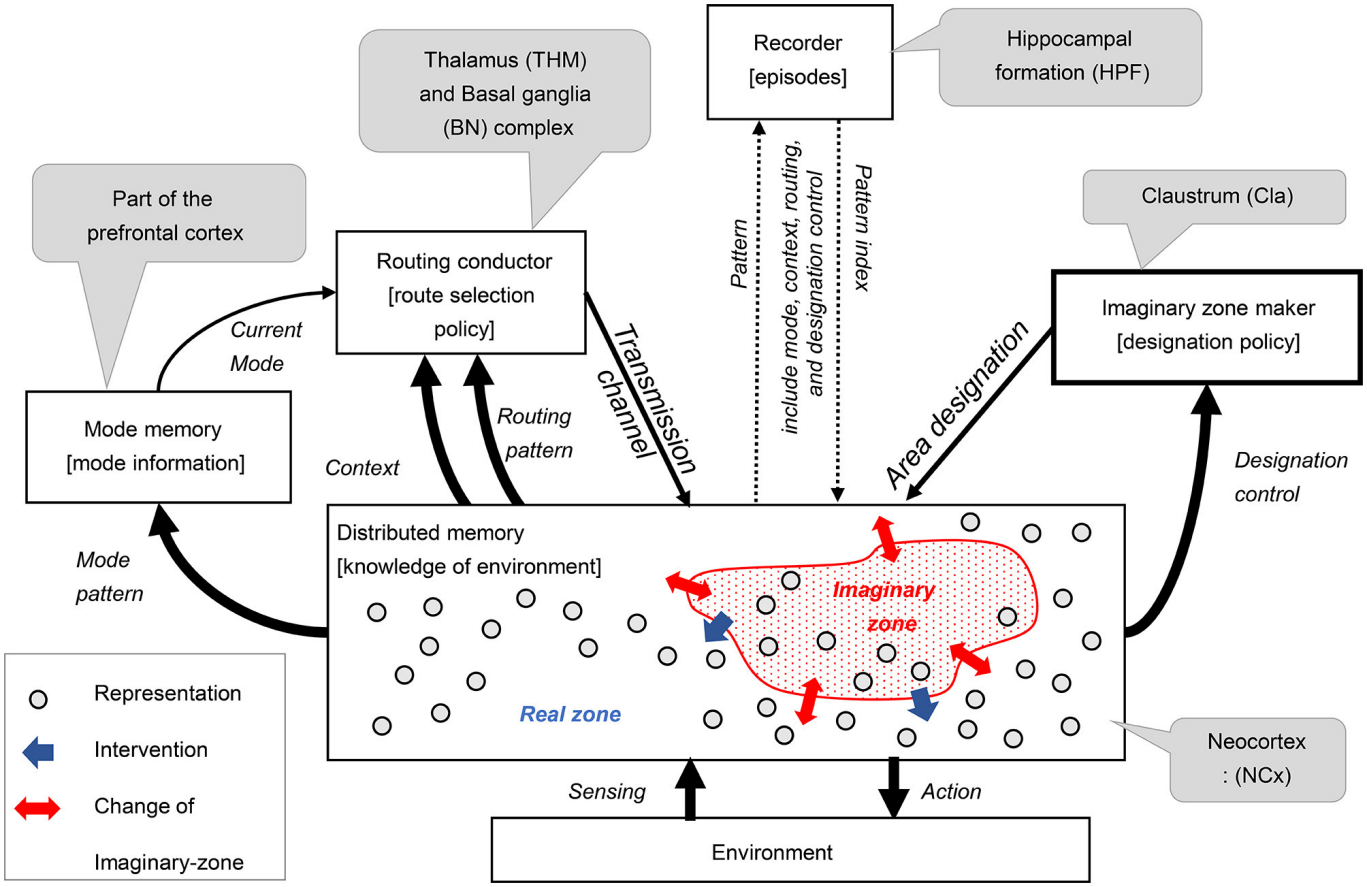
Architecture proposed for imagination composed with six components.

Figure 3 shows a schematic representation of the brain regions (mainly the neocortex) treated in this study.

#### 3.4.1 Distributed memory is allocated to a neocortex

The distributed memory allocated to the neocortex as a precondition has the following mechanisms, as described in Section 3.2.1. First, imaginary zones can be allocated according to the obtained area designation signals to realize the dynamic zone partitioning function. In this case, the remaining areas are used as a real zone. The RAE function receives the sensing signals in the real zone, processes them in real-time, and outputs them as actions. In contrast, the manipulation function determines the combination of expressions based on the transmission line signals in the imaginary zone. It also has an intervention function that allows patterns imaged in the imaginary zone to intervene in processing the real zone. To further realize the reuse function, it receives a pattern index signal and reproduces the pattern on expression.

In the following, the mechanism of distributed memory, which corresponds to the structure of the neocortex, will be explained, focusing on the functions it performs.

**Figure 4:**
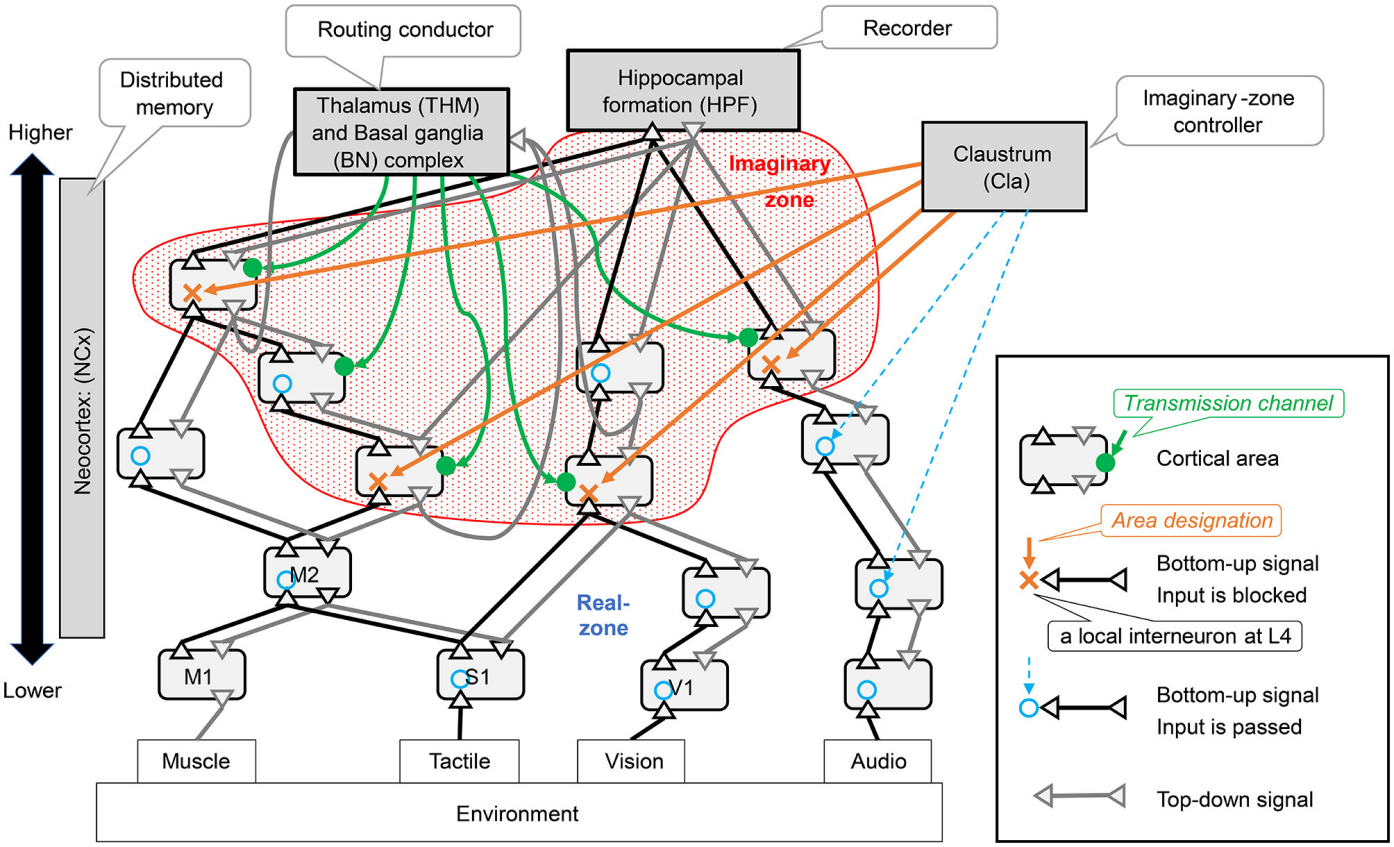
Neocortical schemes and related brain organs: the symbols in parentheses for each brain region are brain region identifiers as defined in the DHBA (Developing Human Brain Atlas) ontology. This study assumes that distributed memory corresponds to the neocortex (ref. Section 3.2.1). The neocortex is located at the center of Figure 3 and consists of many interconnected regions. There are two types of connections between areas: bottom-up pathways that transmit observed signals from the environment to the inside and top-down pathways that transmit signals in the opposite direction.

Dynamic zone partitioning function is realized by a mechanism in the neocortex that blocks the bottom-up observation signals in units smaller than the area. This mechanism involves interneurons exerting an inhibitory effect on layer 4, which receives bottom-up signals. In the Figure 4, the area where the bottom-up signal passes through is indicated by a blue circle, and a red X indicates where it is blocked. In this study, we assumed that the area designation signal to specify the area to be blocked originates from the Claustrum. We hypothesized that when the bottom-up signal is properly blocked in the neocortex, an imaginary zone isolated from observation is formed.

The RAE function is a neocortical mechanism involving a set of areas where the influx of observation signals other than those formed by the imaginary zone above is not blocked; thus, we refer to this area as the real zone. The real zone is a mechanism that processes Sensing signals from the environment in real-time and outputs the results as Actions to the environment. When performing this processing, the routing conductor mechanism can be used to bind different types of information.

The manipulation function is a mechanism that receives coherent low-frequency oscillation signals, which are transmission channel signals, from multiple domains, and transmits high-frequency oscillations modulated by Phase-Amplitude Coupling between those domains by Cortical Dynamic Routing. This mechanism allows it to imagine various images.

Intervening functions require mechanisms by which imagined patterns in imaginary zones affect processing in real zones. The anatomical projection patterns between laminar structures across domains have not yet been fully elucidated in corticocortical connections. However, there is a large body of knowledge regarding top-down corticocortical connections from higher-order to lower-order domains (Rockland and Pandya, 1979; Felleman and Van Essen, 1991; Barbas et al., 2005; Medalla and Barbas, 2006; Shipp, 2007; Solari and Stoner, 2011; Markov et al., 2014; Barbas, 2015). Thus, assuming that the intervention function is realized through those top-down connections is not unnatural.

The history management function seems to be realized in the hippocampus, as discussed in Section 3.4.5 below. The hippocampus can consolidate relatively higher-order activity patterns from the widely dispersed memories of the neocortex. Moreover, episodic memories stored in the hippocampus are known to be reproduced in the neocortex; however, the specific details of this mechanism are unknown. Therefore, it is reasonable to assume that the neocortex also has a mechanism for reproducing patterns in its representation based on received pattern-index signals.

#### 3.4.2 Imaginary zone maker is allocated to claustrum

The brain ROI corresponding to the imaginary zone maker mechanism can be considered a claustrum. This is because the inputs (designation control signals) and outputs (area designation signals) of the imaginary zone-maker mechanism can be mapped to the inputs and outputs of the claustrum. The output of area designation signals may be able to control the blockage of the aforementioned bottom-up input by projection from pyramidal cells within the claustrum to inhibitory neurons in layer 4 of the neocortex; this occurs in both the sensory and motor cortex. This has been termed the Gain Control Signal of Sensory Processing in previous studies (Goll et al., 2015). It is reasonable to assume that the designated control signal that controls the behavior of the imaginary zone maker is a projection from some higher-order areas. In fact, there is a reciprocal projection from the frontal lobes (Goll et al., 2015), which supports this validity. As a result, because the frontal disabilities correspond to components of the imaginary zone maker, their function is dynamic zone partitioning.

#### 3.4.3 Routing conductor is allocated to the complex of thalamus and basal ganglia

The brain ROI responsible for the routing conductor mechanism is a complex consisting of the basal ganglia and thalamus. This is because the inputs (current mode signals, context signals, and routing pattern signals) and outputs (transfer path signals) of the routing conductor mechanism can be naturally mapped to the inputs and outputs of that complex.

The inputs of the current mode and Context signals correspond to the striatum of the basal ganglia, which can receive information from various areas of the neocortex. The input of the Routing Pattern signal can correspond to the thalamus (matrix-type relay cells), which receives information from various areas of the neocortex. As the core-type relay cells in the thalamus are thought not to be controlled by the basal ganglia, we focused on the matrix-type relay cells. The output of transmission channel signals corresponds to a mechanism in which the thalamus (matrix-type relay cells) sends the same low-frequency oscillatory signal to L1 in the neocortex for the transmission channel it wishes to include in the exchange of information between representations, establishing interregional transmission channels L2/3/4.

The route selection policy within the complex can be considered to be realized by a mechanism in which the internal globus pallidus (GPi)/substantia nigra pars reticulata (SNr) nuclei, the output of reinforcement learning in the basal ganglia, disinhibit the thalamus (matrix relay cells).

From the above, the input/output signals required for the routing conductor mechanism can be semantically mapped to the input/output of a complex consisting of the basal ganglia and thalamus. Therefore, this complex can be considered as the ROI of the brain circuitry responsible for the routing conductor mechanism. The TLF corresponding to this ROI is the manipulation function defined in Step 2.

#### 3.4.4 Mode memory is allocated to a specific agranular neocortical area

In this section, we explain that the ROI responsible for the Mode memory mechanism in the brain is likely to be one of the agranular higher neocortical areas.

The mode memory mechanism refers to the mode pattern signals from the distributed memory to generate, maintain, and send out the current mode signals used to adjust the routing conductor's route selection policy. As previously mentioned in Section 2.1, modes are diverse, including present/past/future, self/other, and belief/intention. In neuroscience, prospective memory, including temporal modes (Owen, 1997; Okuda et al., 1998, 2007; Tanji and Hoshi, 2008; Reynolds et al., 2009) associated with activity in the rostral PFC (A10). Moreover, studies of facial apraxia have shown temporal lobe involvement (Gainotti and Marra, 2011). Given these examples, the modal pattern signals representing the mode are likely to reside in multiple distributed locations in the neocortex. However, the Routing conductor must operate based on a specific mode at a given time, and sustain that operation for a certain period. Thus, the mode storage mechanism focuses on specific mode patterns in the distributed memory and maintains the mode to be processed.

At this stage, identifying the specific neocortical areas corresponding to the memory mode is challenging. However, the area is assumed to be located in a higher-order region unrelated to any sensor or behavior. Furthermore, the modal memory itself is real and does not need to distinguish between imagination. Therefore, an agranular neocortical area with no granular layer to block input would be appropriate. From this point of view, areas such as the anterior cingulate cortex (MFC) are promising areas for mode memory.

#### 3.4.5 Recorder is allocated to hippocampal formation

The ROI responsible for the Recorder mechanism in the brain corresponds to the hippocampal formation (HPF), which is composed of the hippocampus and the entorhinal cortex (EC).

First, as an internal mechanism of the Recorder, it acquires pattern signals from various parts of distributed memory, records them as pattern indexes, and outputs pattern index signals to reproduce patterns in the allocated memory. A pattern signal is an overall representation in distributed memory that includes various patterns such as mode, context, routing, designated control, etc. In the hippocampus, the dentate gyrus, which receives input from EC, isolates patterns (Kesner, 2007), and the recurrent circuits of CA3 and CA2 that receive this information can rapidly encode the sequence of those patterns (Rolls et al., 2005; Kesner, 2007; Kubik et al., 2007; Rolls, 2007; Behrendt, 2013; Okamoto and Ikegaya, 2019). This configuration is thought to allow the hippocampus to associate and encode various information conveyed by input from EC to form temporally linked episodic memories (Fortin et al., 2004; Eichenbaum and Fortin, 2005) and participate in episodic replay along with information about time and place (Ergorul and Eichenbaum, 2004). Thus, the internal mechanisms of the recorder would be consistent with HPF.

Furthermore, for the Recorder to perform the function of history management concerning distributed memory, it must be signal interconnected with distributed memory, acquire pattern signals over a wide area, and output a pattern index. The hippocampus presents structures that interconnect with extensive areas of the neocortex via EC, and this structure is conserved across species (Eichenbaum, 2000; Vincent et al., 2006; Kahn et al., 2008; Behrendt, 2013). Therefore, the connection form of recorders and distributed memory may correspond to the form of HPF and neocortex.

From the above discussion, it seems reasonable to map the Recorder mechanism to HPF regarding both internal mechanisms and connection form.

### 3.5 Summary of the hypothesis of imagination architecture

The results provide the hypothesis that imaginary zone makers map to the claustrum, routing conductors to a complex consisting of the basal ganglia and thalamus, mode memory to a specific agranular neocortical area, and recorders to HPF. The above finding of multifaceted concordance between the internal processing of the components and the meaning of the signals exchanged would seem to validate this hypothesis. The observed multifaceted concordance between the internal functioning of these components and the semantic nature of the exchanged signals lends considerable support to this hypothesis.

Table 1 shows the function of each component and the corresponding brain organ, including the mapping of distributed memory to the neocortex, which is the premise of this study. Table 2 then shows the signals exchanged between those components and their functional meaning.

**Table 1:** Components of imagination architecture.

**Functions**	**Representation and execution RAE**	**Dynamic-zone partitioning**	**Manipulation**	**Mode maintenance**	**History management**
Description of functions	Functions to represent different types of information. There are sensory representations to execute perception, motor representations to execute control, and higher-order representations to execute thought. These functions can be divided into those that output actions in real-time to the environment and those that are imaginative and do not contribute directly to actions. However, functions that reflect the results of imagination in real-time behavior must also be included to meet intervention requirements.	This function dynamically specifies an imaginary zone in distributed memory and maintains it for a necessary period. An imaginary zone is a subset of the representation for executing imagination on distributed memory. This functionality allows for manipulation and flexible imagination requirements.	This function imagines new patterns by manipulating representations on the distributed memory according to a policy appropriate to its type of mode and context to meet the requirements of manipulation and modal control.	This function maintains the current mode that affects the sequence control of patterns generated or replayed on the distributed memory to meet the requirements of mode control.	This function records the pattern of activity in distributed memory at a given time as an episode. It restores patterns of activity in distributed memory from episodes as needed. Patterns include mode, context, routing, and designation control.
**Components**	**Distributed memory**	**Imaginary zone maker**	**Routing conductor**	**Mode memory**	**Recorder**
Mechanism of components	This component is a distributed memory component is a highly versatile, distributed associative storage system that excels at representing and manipulating various patterns. This component outputs real-time action signals in response to environmental sensing signals. The various internal pattern information is distributed to the surrounding components. This component dynamically sets imaginary zones based on area designation signals, changes associative behavior according to transmission channel signals, and reproduces patterns according to pattern index signals.	The designation policy generates area designation signals that specify areas of imaginary zones on distributed memory based on the received designation control signals.	The route selection policy chooses and generates the appropriate transmission channel signals from this component's received routing pattern signals. This selection is controlled according to the type of mode obtained from the current mode signal, depending on the context information by the received context signal.	This component holds the specific mode pattern signal obtained from one of the distributed memory as mode information and transmits it continuously as a current mode signal.	This component records the episodes as indexes of acquired patterns. These episodes restore and send to distributed memory when needed.
**Brain region**	**Neocortex**	**Claustrum**	**The complex of Thalamus and Basal ganglia**	**Specific agranular neocortical area**	**Hippocampal formation (HPF)**
Biological counterpart of input signals	- Sensing: Environment ->Primary sensory cortex (via thalamus) - Area designation: Claustrum ->Sensory / Motor cortexes - Transmission channel: Thalamus matrix type relay neurons ->Layer 1 of the broad area of neocortex - Pattern index: Entorhinal cortex ->Broad area of neocortex	- Designation control: Higher neocortical area ->Claustrum	- Current mode: Specific agranular neocortical area ->Striatum - Context: Neocortex ->Striatum - Routing pattern Broad areas of Neocortex ->Thalamus (matrix-type relay cells)	- Mode pattern: Diverse areas of the neocortex ->Specific agranular neocortical area	- Pattern: Broad areas of Neocortex ->Entorihinal cortex
Biological counterpart of output signals	- Action: Motor cortex ->Muscles - Designation control: Higher neocortical area ->Claustrum - Context: Neocortex ->Striatum - Mode pattern: Diverse areas of the neocortex ->Specific agranular neocortical - Routing pattern: Broad areas of Neocortex ->Thalamus (matrix-type relay cells) - Pattern: Broad areas of Neocortex ->Entorihinal cortex	- Area designation: Claustrum ->Layer 4 of Sensory/Motor cortexes	- Transmission channel: Thalamus (matrix-type relay cells) ->Layer 1 of the broad area of neocortex	- Current mode: Specific agranular neocortical area ->Striatum	- Pattern index: Entorhinal cortex ->Broad area of Neocortex
Biological counterpart of internal mechanisms	In the neocortex, the anatomical regions in which specific types of information are represented and stored (with some plasticity) are generally consistent across individuals. It is therefore reasonable to view the neocortex as a distributed memory that is loosely modular.	The claustrum is well suited for this mechanism, as it projects to the neocortex's fourth layer of the sensory and motor cortices, where it can block observational input from the outside world.	The pathway selection policy within the complex can be achieved by a mechanism in which the GPi/SNr nucleus, the output of reinforcement learning in the basal ganglia, de-represses the thalamus (matrix-type relay cells)	Since the modal memory is not supposed to be fictional, it does not need to be gated by the granular layer. Thus, specific agranular neocortical areas would provide this mechanism.	With its internal mechanisms to store and reproduce episodes and structures that interconnect with extensive areas of the neocortex, HPF is well suited for this mechanism.

**Table 2:** Signals of the imagination architecture.

**Signal name**	**Source**	**Target**	**Signal semantics**
Action	Motor cortex	Muscles	Behavioral signals output to the environment
Area designation	Claustrum	Sensory/Motor cortexes	Signal that directs the blocking of observation signals to the Imaginary zone area.
Context	Neocortex	Striatum	Signals containing the various contexts used for routing control
Current mode	Specific agranular neocortical area	Striatum of basal ganglia	Mode signals that determine the direction of routing control
Designation control	Higher neocortical area	Claustrum	Command signals that specify the range of the imaginary zone on distributed memory
Mode pattern	Diverse areas of the neocortex	Specific agranular neocortical	Signals that contain information about a specific mode
Pattern	Broad areas of Neocortex	Entorhinal cortex	Any pattern on the neocortex, including mode, context, routing, and specified control information
Pattern index	Entorhinal cortex	Broad area of neocortex	Signals of indices for various patterns appeared on distributed memory
Routing pattern	Broad areas of Neocortex	Thalamus (matrix-type relay cells)	Signals represent patterns in the routing of signals exchanged among areas.
Sensing	Environment	Primary sensory cortex (via thalamus)	Sensor signals obtained from the environment
Transmission channel	Thalamus (matrix-type relay cells)	L1 of the broad area of neocortex	Signals that specify the exchanging channels among multiple areas on distributed memory

## 4 Discussions

### 4.1 Brain-constrained architectural design for general-purpose software

In the quest to achieve imagination architecture—the primary focus of this study-it is plausible that a variety of architectural designs could yield the desired outcomes. However, when the objective is to create a system endowed with a functional diversity comparable to human intelligence, the range of viable general-purpose architectures is anticipated to be limited. Under these circumstances, we posit that brain architecture offers at least one feasible solution. Accordingly, we adopt a BRA-driven development approach with the aim of crafting general-purpose, brain-inspired software.

In line with this perspective, we adopt the methodology delineated by Marques and Holland (2009), as referenced in the introduction to this paper. Consequently, our architectural design adheres to the initial three steps of the function-oriented SCID methodology. It should be noted that the fourth step, which involves “brain region mapping,” is not included in their study. As a result, while that architecture may be well-suited for tasks involving imagination, challenges are expected to arise when integrating this architecture with other diverse capabilities.

### 4.2 Assigning functional roles to the claustrum and thalamus

In this study, within an architectural framework inspired by neurobiology, specified specific functional roles for two prominent brain regions that have inter-projective relationships with broader areas of the neocortex. Specifically, the role of the Imaginary Zone Maker has been attributed to the claustrum, while the function of the Routing Conductor has been allocated to the thalamus.

Subsequently, the anatomical rationale for these targeted functional allocations is elucidated as follows:

- **Complexity of the assigned functions:** The computational complexity of routing inter-regional information is proportional to the combinatorial array of areas implicated. Conversely, the computational demands associated with establishing Imaginary Zones are essentially linear concerning the number of areas involved. Given the thalamus's anatomical size compared to the claustrum (Smith et al., 2020), it is judicious to assign the computationally demanding role of the Routing Conductor to the thalamus.
- **Modulation of neocortical layer activity and inter-area routing:** Matrix relay cells in the thalamus project to the first layer of the neocortex (Jones, 1998; Rodriguez et al., 2004; Bonjean et al., 2012; Kaneko, 2013; Harris and Shepherd, 2015; Wei et al., 2016), a feature that renders them optimally positioned to modulate neuronal activity in the superficial layers of the neocortex. That projection is suited for routing inter-area communication pathways within the supra-granular layers of the neocortex.
- **Information filtering and the establishment of imaginary zones:** The claustrum is demonstrably well-adapted to fulfill the role of Imaginary Zone Maker, a function that necessitates the attenuation or blockage of bottom-up informational flow. This specific functionality demands a broad projection to the fourth layer of the neocortex, where the requisite information may be effectively intercepted. Although core-type relay cells in the thalamus do indeed project to this neocortical layer, the relatively limited spatial extent of each neuron's projection (Bonjean et al., 2012) hampers their efficacy for this particular role.

### 4.3 Future directions and iterative improvement of imagination architecture

In future work, we will utilize the brain structures identified in this study as Regions of Interest (ROIs) and their corresponding functions as Top-Level Functions (TLFs) (see Tables 1, 2) in the conventional SCID method. This approach will enable more detailed reverse engineering of the computational capabilities of each component.

As an example of applying the SCID method to individual TLFs, we can revisit the role of the claustrum in our imagination architecture. In the Section 3.4.2, we assigned the function of Imaginary Zone Maker to the claustrum. However, recent research suggests that its role may be more complex and multifaceted. Reviews by Jackson et al. (2020) and Madden et al. (2022) highlight various hypotheses regarding the function of the claustrum, including its involvement in saliency processing, attention allocation, and consciousness. Additionally, a recent study (McBride et al., 2023) demonstrates that the effects of claustrum-to-cortex projections vary depending on the target brain region, cortical layer, and cell type. These findings suggest potential avenues for future research to refine our understanding of the claustrum's role in imagination.

This detailed examination of the claustrum serves as a template for how we intend to approach the study of other brain organs involved in our Imagination Architecture. Building on this approach, we aim to develop more sophisticated architectural designs for each brain organ by incorporating additional neuroanatomical details. This will include consideration of the strength of projections (connections) between regions and the laminar origin of these projections. By applying the SCID method to each ROI and its corresponding TLF, we hope to create models that better reflect the intricate structure and function of each brain organ's role in imaginative processes.

As we progress with the detailed application of the SCID method to individual brain organs, starting with the claustrum and extending to others such as the thalamus, basal ganglia, and hippocampal formation, we anticipate that new discoveries may emerge. These findings could potentially feed back into and refine our overall Imagination Architecture. For instance, a more nuanced understanding of the claustrum's function might lead to adjustments in how we conceptualize the Imaginary Zone Maker component. Similarly, new insights into the thalamus-basal ganglia complex could refine our understanding of the Routing Conductor component. This feedback process would involve reassessing the roles and interactions of components based on new neuroanatomical and functional data, potentially leading to the addition, modification, or even merging of components in our architecture.

This iterative process represents a crucial next step in our research. Applying conventional SCID methods to the ROIs and TLFs identified in this study will allow us to delve deeper into the specific computational mechanisms underlying each component of our imagination architecture. This more granular analysis will likely reveal additional subtleties and complexities that can further inform and refine our model. By systematically incorporating these insights, we aim to develop a more comprehensive and biologically authentic representation of the brain's imaginative capabilities. This enhanced model would not only more accurately reflect the intricate workings of the brain but also provide a stronger foundation for future computational models of imagination and related cognitive processes.

## 5 Conclusions

In this study, we introduced the function-oriented SCID method, which overcomes the limitation of the existing SCID method applying only to a narrow range of the brain. This method enables reverse engineering across a broader spectrum of brain regions. The approach consists of capability and requirements definition (Step 1), function decomposition (Step 2), architecture design (Step 3), and brain region mapping (Step 4). If the mapping in Step 4 is unsuccessful, we return to Step 2 to redesign the decomposition into independent functions.

Subsequently, we applied the function-oriented SCID method to the imagination, which plays a central role in human intelligence. This is because imagination necessitates the coordination of various brain organs, such as the neocortex, basal ganglia, thalamus, and hippocampus.

In the proposed architecture, the Distributed memory component associated with the Neocortex realizes the RAE function; the Imaginary zone maker component associated with the Claustrum accomplishes the Dynamic-zone partitioning function; the Routing conductor component linked with the Complex of Thalamus and Basal ganglia performs the Manipulation function; the Mode memory component related to the Specific agranular neocortical area executes the Mode maintenance function; and the Recorder component affiliated with the Hippocampal formation handles the History management function.

Our methodology thoroughly accounts for the requirements needed to realize imagination in the brain, leading to a functionally decomposed, biologically plausible architecture. However, because we focus on a single, abstract function-imagination-the range of possible hypotheses is not narrow enough. As a result, it's challenging to eliminate the designer's arbitrary factors from the architectural design process.

### 5.1 Implications and applications

The proposed brain-consistent architecture for imagination has several implications and potential applications across various fields:

- **Artificial intelligence**: The architecture can provide insights for developing AI systems with imaginative capabilities. Since the 2010s, AI has seen significant progress in imagination-related technologies, particularly around world models (Ha and Schmidhuber, 2018; Hafner et al., 2019). Some of these research efforts have also considered the relationship between AI architectures and brain architectures (Friston et al., 2021; Taniguchi et al., 2021; Safron, 2022). By incorporating the key components and their interactions outlined in the proposed brain-consistent architecture, researchers may be able to create AI agents that can generate novel ideas, simulate future scenarios, and adapt to unfamiliar situations, potentially leading to more creative and flexible AI systems in areas such as problem-solving, decision-making, and artistic creation.
- **Cognitive psychology**: The proposed architecture offers a framework for understanding the cognitive processes underlying imagination. It can guide further research into the roles of different brain regions and their interactions in imaginative thought. This could provide insights into individual differences in imaginative abilities and how they relate to other cognitive functions such as memory, perception, and reasoning.
- **Neuroscience**: The mapping of architectural components to specific brain regions can stimulate targeted investigations into the neural mechanisms of imagination. Researchers can use the proposed architecture to generate testable hypotheses about the functions of the claustrum, thalamus, and other implicated brain areas. This could advance our understanding of how the brain generates and manipulates mental representations.
- **Mental health**: Understanding the neural basis of imagination could potentially contribute to the development of interventions for disorders that involve impairments in imaginative abilities. The proposed architecture could guide the identification of specific neural targets for therapeutic interventions and the design of cognitive training programs to enhance imaginative skills.
- **Education**: Insights from the proposed architecture could be applied to educational practices that aim to foster creativity and imaginative thinking. By understanding the cognitive and neural mechanisms involved in imagination, educators can design learning activities and environments that optimize the development of these abilities in students across various domains, from the arts to the sciences.

In conclusion, this study's brain-consistent architecture for imagination has wide-ranging implications and applications. It can contribute to advancements in artificial intelligence, cognitive psychology, neuroscience, mental health, and education. By providing a comprehensive framework for understanding the neural basis of imagination, this work opens up new avenues for research and development in these diverse fields.

## Data availability statement

The original papers presented in this study are included in the paper, further inquiries can be directed to the corresponding author.

## Ethics statement

Ethical approval was not required for the study involving animals in accordance with the local legislation and institutional requirements because this study is a knowledge-integrated study based entirely on the findings of existing publications. Therefore, ethical issues such as animal experimentation cannot arise.

## Author contributions

HY: Conceptualization, Data curation, Formal analysis, Investigation, Methodology, Project administration, Visualization, Writing – original draft, Writing – review & editing. AF: Conceptualization, Methodology, Validation, Writing – original draft, Writing – review & editing. IY: Supervision, Validation, Writing – review & editing. YM: Funding acquisition, Resources, Supervision, Writing – review & editing.

## Footnotes

^1^The term index is used here because there is a theory (Teyler and Rudy, 2007) that the hippocampus, which is assumed to be responsible for this function in the brain, has an index that specifies a representation in distributed memory. In deep learning research, this feature has been modeled as a generative mechanism (Shin et al., 2017).

## References

[B1] Agnati L. F. Guidolin D. Battistin L. Pagnoni G. Fuxe K. (2013). The neurobiology of imagination: possible role of interaction-dominant dynamics and default mode network. Front. Psychol. 4:296. 10.3389/fpsyg.2013.00296 23745117 PMC3662866

[B2] Baars B. J. (1993). A Cognitive Theory of Consciousness. Cambridge: Cambridge University Press.

[B3] Baars B. J. Franklin S. Ramsoy T. Z. (2013). Global workspace dynamics: Cortical “binding and propagation” enables conscious contents. Front. Psychol. 4:200. 10.3389/fpsyg.2013.00200 23974723 PMC3664777

[B4] Barbas H. (2015). General cortical and special prefrontal connections: principles from structure to function. Annu. Rev. Neurosci. 38, 269–289. 10.1146/annurev-neuro-071714-033936 25897871

[B5] Barbas H. Hilgetag C. C. Saha S. Dermon C. R. Suski J. L. (2005). Parallel organization of contralateral and ipsilateral prefrontal cortical projections in the rhesus monkey. BMC Neurosci. 6:32. 10.1186/1471-2202-6-32 15869709 PMC1134662

[B6] Behrendt R.-P. (2013). Conscious experience and episodic memory: hippocampus at the crossroads. Front. Psychol. 4:304. 10.3389/fpsyg.2013.00304 23755033 PMC3667233

[B7] Bonjean M. Baker T. Bazhenov M. Cash S. Halgren E. Sejnowski T. (2012). Interactions between core and matrix thalamocortical projections in human sleep spindle synchronization. J. Neurosci. 32, 5250–5263. 10.1523/JNEUROSCI.6141-11.2012 22496571 PMC3342310

[B8] Bratman M. Others (1987). Intention, Plans, and Practical Reason, volume 10. Cambridge, MA: Harvard University Press.

[B9] Chang A. Y. C. Biehl M. Yu Y. Kanai R. (2019). Information closure theory of consciousness. Front. Psychol. 11:1504. 10.3389/fpsyg.2020.01504 32760320 PMC7374725

[B10] Craig J. Baron-Cohen S. (1999). Creativity and imagination in autism and asperger syndrome. J. Autism Dev. Disord. 29, 319–326. 10.1023/A:1022163403479 10478731

[B11] Crespi B. Leach E. Dinsdale N. Mokkonen M. Hurd P. (2016). Imagination in human social cognition, autism, and psychotic-affective conditions. Cognition 150, 181–199. 10.1016/j.cognition.2016.02.001 26896903

[B12] Crespi B. J. (2016). Autism as a disorder of high intelligence. Front. Neurosci. 10:300. 10.3389/fnins.2016.00300 27445671 PMC4927579

[B13] Crick F. Koch C. (2003). A framework for consciousness. Nat. Neurosci. 6, 119–126. 10.1038/nn0203-119 12555104

[B14] Crick F. C. Koch C. (2005). What is the function of the claustrum? Philos. Trans. R. Soc. Lond. B. Biol. Sci. 360, 1271–1279. 10.1098/rstb.2005.1661 16147522 PMC1569501

[B15] di Pellegrino G. Fadiga L. Fogassi L. Gallese V. Rizzolatti G. (1992). Understanding motor events: a neurophysiological study. Exp. Brain Res. 91, 176–180. 10.1007/BF00230027 1301372

[B16] Diamond J. (1992). The third chimpanzeee: the evolution and future of the human animal. Oneworld Publications. 10.1002/em.2850200411

[B17] Duch W. (2007). Intuition, insight, imagination and creativity. IEEE Comput. Intell. Magaz. 2, 40–52. 10.1109/MCI.2007.385365

[B18] Edelman G. M. Tononi G. (2000). “Reentry and the dynamic core,” in Neural Correlates of Consciousness, ed. T. Metzinger (Lonton: The MIT Press), 139–151. 10.7551/mitpress/4928.003.0012

[B19] Eichenbaum H. (2000). A cortical-hippocampal system for declarative memory. Nat. Rev. Neurosci. 1, 41–50. 10.1038/35036213 11252767

[B20] Eichenbaum H. Fortin N. J. (2005). Bridging the gap between brain and behavior: cognitive and neural mechanisms of episodic memory. J. Exp. Anal. Behav. 84, 619–629. 10.1901/jeab.2005.80-04 16596982 PMC1389783

[B21] Ergorul C. Eichenbaum H. (2004). The hippocampus and memory for “what,” “where,” and “when”. Learn. Mem. 11, 397–405. 10.1101/lm.73304 15254219 PMC498318

[B22] Felleman D. J. Van Essen D. C. (1991). Distributed hierarchical processing in the primate cerebral cortex. Cereb. Cortex 1, 1–47. 10.1093/cercor/1.1.1 1822724

[B23] Fortin N. J. Wright S. P. Eichenbaum H. (2004). Recollection-like memory retrieval in rats is dependent on the hippocampus. Nature 431, 188–191. 10.1038/nature02853 15356631 PMC4053162

[B24] Friston K. Da Costa L. Hafner D. Hesp C. Parr T. (2021). Sophisticated inference. Neural Comput. 33, 713–763. 10.1162/neco_a_01351 33626312

[B25] Gainotti G. Marra C. (2011). Differential contribution of right and left temporo-occipital and anterior temporal lesions to face recognition disorders. Front. Hum. Neurosci. 5:55. 10.3389/fnhum.2011.00055 21687793 PMC3108284

[B26] Garson J. (2018). Modal Logic. Metaphysics Research Lab, Stanford University, fall 2018 edition.

[B27] Goll Y. Atlan G. Citri A. (2015). Attention: the claustrum. Trends Neurosci. 38, 486–495. 10.1016/j.tins.2015.05.006 26116988

[B28] Ha D. R. Schmidhuber J. (2018). “Recurrent world models facilitate policy evolution,” in Advances in Neural Information Processing Systems, 2455–2467.

[B29] Hafner D. Lillicrap T. Ba J. Norouzi M. (2019). Dream to control: Learning behaviors by latent imagination. arXiv preprint arXiv:1912.01603.37999968

[B30] Harris K. D. Shepherd G. M. G. (2015). The neocortical circuit: themes and variations. Nat. Neurosci. 18, 170–181. 10.1038/nn.3917 25622573 PMC4889215

[B31] Ichikawa J. Jarvis B. (2012). Rational imagination and modal knowledge. Nous 46, 127–158. 10.1111/j.1468-0068.2010.00811.x

[B32] Ishai A. Sagi D. (1995). Common mechanisms of visual imagery and perception. Science 268, 1772–1774. 10.1126/science.7792605 7792605

[B33] Jackson J. Smith J. B. Lee A. K. (2020). The anatomy and physiology of Claustrum-Cortex interactions. Ann. Rev. Neurosci. 43, 231–247. 10.1146/annurev-neuro-092519-101637 32084328

[B34] James W. (1890). The Principles of Psychology. London: Williams and Norgate. 10.1037/10538-000

[B35] Johnson M. (2013). The Body in the Mind: The Bodily Basis of Meaning, Imagination, and Reason. Chicago: University of Chicago Press.

[B36] Jones E. G. (1998). Viewpoint: the core and matrix of thalamic organization. Neuroscience 85, 331–345. 10.1016/S0306-4522(97)00581-2 9622234

[B37] Jung R. E. Flores R. A. Hunter D. (2016). A new measure of imagination ability: anatomical brain imaging correlates. Front. Psychol. 7, 496. 10.3389/fpsyg.2016.00496 27148109 PMC4834344

[B38] Kahn I. Andrews-Hanna J. R. Vincent J. L. Snyder A. Z. Buckner R. L. (2008). Distinct cortical anatomy linked to subregions of the medial temporal lobe revealed by intrinsic functional connectivity. J. Neurophysiol. 100, 129–139. 10.1152/jn.00077.2008 18385483 PMC2493488

[B39] Kaneko T. (2013). Local connections of excitatory neurons in motor-associated cortical areas of the rat. Front. Neural Circ. 7:75. 10.3389/fncir.2013.00075 23754982 PMC3664775

[B40] Kesner R. P. (2007). Behavioral functions of the CA3 subregion of the hippocampus. Learn. Mem. 14, 771–781. 10.1101/lm.688207 18007020

[B41] Kind A. (2016). The Routledge Handbook of Philosophy of Imagination. London: Routledge. 10.4324/9781315657905

[B42] Kinsbourne M. (1988). “An integrated field theory of consciousness,” in Consciousness in Contemporary Science, eds. A. J. Marcel, and E. Bisiach (Oxford: Oxford University Press).

[B43] Kubik S. Miyashita T. Guzowski J. F. (2007). Using immediate-early genes to map hippocampal subregional functions. Learn. Mem. 14, 758–770. 10.1101/lm.698107 18007019

[B44] Kung P. (2010). Imagining as a guide to possibility. Philos. Phenomenol. Res. 81, 620–663. 10.1111/j.1933-1592.2010.00377.x

[B45] Lee S. Parthasarathi T. Kable J. W. (2021). The ventral and dorsal default mode networks are dissociably modulated by the vividness and valence of imagined events. J. Neurosci. 41, 5243–5250. 10.1523/JNEUROSCI.1273-20.2021 34001631 PMC8211541

[B46] Liao S.-Y. Gendler T. (2020). Imagination. Metaphysics Research Lab, Stanford University, summer 2020 edition.

[B47] Llinás R. Ribary U. Contreras D. Pedroarenan C. (1998). The neuronal basis for consciousness. Philos. Trans. R. Soc. London 353, 1841–1849. 10.1098/rstb.1998.0336 9854256 PMC1692417

[B48] Madden M. B. Stewart B. W. White M. G. Krimmel S. R. Qadir H. Barrett F. S.. (2022). A role for the claustrum in cognitive control. Trends Cogn. Sci. 26, 1133–1152. 10.1016/j.tics.2022.09.006 36192309 PMC9669149

[B49] Markov N. T. Vezoli J. Chameau P. Falchier A. Quilodran R. Huissoud C.. (2014). Anatomy of hierarchy: feedforward and feedback pathways in macaque visual cortex. J. Comp. Neurol., 522, 225–259. 10.1002/cne.23458 23983048 PMC4255240

[B50] Marques H. G. Holland O. (2009). Architectures for functional imagination. Neurocomputing 72, 743–759. 10.1016/j.neucom.2008.06.016

[B51] McBride E. G. Gandhi S. R. Kuyat J. R. Ollerenshaw D. R. Arkhipov A. Koch C.. (2023). Influence of claustrum on cortex varies by area, layer, and cell type. Neuron 111, 275–290.e5. 10.1016/j.neuron.2022.10.026 36368317

[B52] McFadden J. (2002). Synchronous firing and its influence on the brain's electromagnetic field. J. Consc. Stud. 9, 23–50.23391515

[B53] Medalla M. Barbas H. (2006). Diversity of laminar connections linking periarcuate and lateral intraparietal areas depends on cortical structure. Eur. J. Neurosci. 23, 161–179. 10.1111/j.1460-9568.2005.04522.x 16420426

[B54] Nichols S. (2006). The Architecture of the Imagination: New Essays on Pretence, Possibility, and Fiction. Oxford: Clarendon Press. 10.1093/acprof:oso/9780199275731.001.000136389024

[B55] Nichols S. Stich S. P. (2003). Mindreading: An Integrated Account of Pretence, Self-Awareness, and Understanding Other Minds. Oxford: Oxford University Press. 10.1093/0198236107.001.0001

[B56] Okamoto K. Ikegaya Y. (2019). Recurrent connections between CA2 pyramidal cells. Hippocampus 29, 305–312. 10.1002/hipo.23064 30588702

[B57] Okuda J. Fujii T. Ohtake H. Tsukiura T. Yamadori A. Frith C. D.. (2007). Differential involvement of regions of rostral prefrontal cortex (brodmann area 10) in time- and event-based prospective memory. Int. J. Psychophysiol. 64, 233–246. 10.1016/j.ijpsycho.2006.09.009 17126435

[B58] Okuda J. Fujii T. Yamadori A. Kawashima R. Tsukiura T. Fukatsu R.. (1998). Participation of the prefrontal cortices in prospective memory: evidence from a PET study in humans. Neurosci. Lett. 253, 127–130. 10.1016/S0304-3940(98)00628-4 9774166

[B59] Owen A. M. (1997). Cognitive planning in humans: neuropsychological, neuroanatomical and neuropharmacological perspectives. Progr. Neurobiol. 53, 431–450. 10.1016/S0301-0082(97)00042-7 9421831

[B60] Pearson J. (2019). The human imagination: the cognitive neuroscience of visual mental imagery. Nat. Rev. Neurosci. 20, 624–634. 10.1038/s41583-019-0202-9 31384033

[B61] Pearson J. Clifford C. W. G. Tong F. (2008). The functional impact of mental imagery on conscious perception. Curr. Biol. 18, 982–986. 10.1016/j.cub.2008.05.048 18583132 PMC2519957

[B62] Perner J. (1991). Understanding the representational mind. Lear. Dev. Concept. Change 12:348.

[B63] Pockett S. (2000). The Nature of Consciousness: A Hypothesis. New York: iUniverse.

[B64] Reynolds J. R. West R. Braver T. (2009). Distinct neural circuits support transient and sustained processes in prospective memory and working memory. Cereb. Cortex 19, 1208–1221. 10.1093/cercor/bhn164 18854581 PMC2665160

[B65] Rockland K. S. Pandya D. N. (1979). Laminar origins and terminations of cortical connections of the occipital lobe in the rhesus monkey. Brain Res. 179, 3–20. 10.1016/0006-8993(79)90485-2 116716

[B66] Rodriguez A. Whitson J. Granger R. (2004). Derivation and analysis of basic computational operations of thalamocortical circuits. J. Cogn. Neurosci. 16, 856–877. 10.1162/089892904970690 15200713

[B67] Rolls E. T. (2007). An attractor network in the hippocampus: theory and neurophysiology. Learn. Mem. 14, 714–731. 10.1101/lm.631207 18007016

[B68] Rolls E. T. Xiang J. Franco L. (2005). Object, space, and object-space representations in the primate hippocampus. J. Neurophysiol. 94, 833–844. 10.1152/jn.01063.2004 15788523

[B69] Rosenthal D. M. (1986). Two concepts of consciousness. Philosop. Stud. 49, 329–359. 10.1007/BF00355521

[B70] Safron A. (2022). Integrated world modeling theory expanded: implications for the future of consciousness. Front. Comput. Neurosci. 16:642397. 10.3389/fncom.2022.642397 36507308 PMC9730424

[B71] Shin H. Lee J. K. Kim J. Kim J. (2017). “Continual learning with deep generative replay,” in Advances in Neural Information Processing Systems, eds. I. Guyon, U. V. Luxburg, S. Bengio, H. Wallach, R. Fergus, S. Vishwanathan, et al. (New York: Curran Associates, Inc.), 2990–2999.

[B72] Shin Y. K. Proctor R. W. Capaldi E. J. (2010). A review of contemporary ideomotor theory. Psychol. Bull. 136, 943–974. 10.1037/a0020541 20822210

[B73] Shipp S. (2007). Structure and function of the cerebral cortex. Curr. Biol. 17, R443–R449. 10.1016/j.cub.2007.03.044 17580069

[B74] Smith J. B. Lee A. K. Jackson J. (2020). The claustrum. Curr. Biol. 30, R1401–R1406. 10.1016/j.cub.2020.09.069 33290700

[B75] Solari S. V. H. Stoner R. (2011). Cognitive consilience: primate non-primary neuroanatomical circuits underlying cognition. Front. Neuroanat. 5:65. 10.3389/fnana.2011.00065 22194717 PMC3243081

[B76] Stanovich K. E. (2009). “Distinguishing the reflective, algorithmic, and autonomous minds: is it time for a tri-process theory,” in In Two Minds: Dual Processes and Beyond, 55–88. 10.1093/acprof:oso/9780199230167.003.000336389024

[B77] Stevens C. F. (2005). Crick and the claustrum. 10.1038/4351040a 15973394

[B78] Taniguchi T. Yamakawa H. Nagai T. Doya K. Sakagami M. Suzuki M.. (2021). Whole brain probabilistic generative model toward realizing cognitive architecture for developmental robots. Neural Netw. 150, 293–312. 10.1016/j.neunet.2022.02.026 35339010

[B79] Tanji J. Hoshi E. (2008). Role of the lateral prefrontal cortex in executive behavioral control. Physiol. Rev. 88, 37–57. 10.1152/physrev.00014.2007 18195082

[B80] Teyler T. J. Rudy J. W. (2007). The hippocampal indexing theory and episodic memory: updating the index. Hippocampus 17, 1158–1169. 10.1002/hipo.20350 17696170

[B81] Tononi G. (2004). An information integration theory of consciousness. BMC Neurosci. 5:42. 10.1186/1471-2202-5-42 15522121 PMC543470

[B82] Tooby J. Cosmides L. (2000). “Consider the source: the evolution of adaptations for decoupling and metarepresentations,” in Metarepresentations: A Multidisciplinary Perspective, New York, 53–116. 10.1093/oso/9780195141146.003.0004

[B83] Vincent J. L. Snyder A. Z. Fox M. D. Shannon B. J. Andrews J. R. Raichle M. E.. (2006). Coherent spontaneous activity identifies a hippocampal-parietal memory network. J. Neurophysiol. 96, 3517–3531. 10.1152/jn.00048.2006 16899645

[B84] Wei Y. Krishnan G. P. Bazhenov M. (2016). Synaptic mechanisms of memory consolidation during sleep slow oscillations. J. Neurosci. 36, 4231–4247. 10.1523/JNEUROSCI.3648-15.2016 27076422 PMC4829648

[B85] Wikipedia contributors (2023). Imagination. Available at: https://en.wikipedia.org/wiki/Imagination (accessed August 6, 2024).

[B86] Xie S. Kaiser D. Cichy R. M. (2020). Visual imagery and perception share neural representations in the alpha frequency band. Curr. Biol. 30, 2621-2627.e5. 10.1016/j.cub.2020.04.074 32531274 PMC7342016

[B87] Yamakawa H. (2021). The whole brain architecture approach: accelerating the development of artificial general intelligence by referring to the brain. Neural Netw. 144, 478–495. 10.1016/j.neunet.2021.09.004 34600220

[B88] Zhang H. Xu L. Zhang R. Hui M. Long Z. Zhao X.. (2012). Parallel alterations of functional connectivity during execution and imagination after motor imagery learning. PLoS ONE 7:e36052. 10.1371/journal.pone.0036052 22629308 PMC3356366

